# The Payment of Medical Officers in V.A.D. Hospitals

**Published:** 1917-08-11

**Authors:** 


### The Hospital

The Workers' Newspaper of Administrative Medicine and Institutiona. Life, Administration, National Insurance and Health.

No. 1627, Vol. LXII. SATURDAY, AUGUST 11, 1917. PRICE ONE PENNY

### THE PAYMENT OF MEDICAL OFFICERS IN V.A.D. HOSPITALS.

One of the earlier local manifestations of a patriotic desire to express the civilian's gratitude to the nation's fighting men was the establishment in all parts of the country of V.A.D. hospitals. In the nature of things medical officers were required for these organisations, and local practitioners either volunteered for such positions or were invited to accept them. Here, as in many other directions, the profession did not hesitate to give its time and skill to those who needed help, and the sense of contributing definite service to those who had suffered in the nation's cause was not an unwelcome experience. Time may in some measure have dulled the early enthusiasm. But it is a poor patriotism which flourishes only when the crowd is shouting, and the medical practitioner is for the most part made of sterner stuff. Hence the medical service of the V.A.D. hospitals has been steadily continued, and has been continued on a voluntary basis. So far as we are able to judge, most of the practitioners engaged in the service are content with this arrangement and do not propose to disturb it. But for a reason which will appear immediately, the question of payment has been raised, and once raised some measure of agitation has followed. On the face of it the issue seems a very simple one. The practitioners concerned accepted a certain position on certain definite conditions. If they find that these conditions are no longer possible to them they have only to say so to the authorities charged with the hospital direction. It may be quite reasonable to point out that service given for a few months is one thing, and service extending over several years is quite another thing. The practitioner may afford to offer the one and yet find it impossible to contribute the other. There is nothing in the least discreditable or dishonourable in stating such a position. Once stated it must then rest with the hospital committee either to find some means of paying the doctor a modest fee, or, failing this, to carry on as best they may without his help. The further necessary remark is that what is possible to one practitioner may be impossible to another, and clearly, therefore, in view of the voluntary character of the original arrangement, the case is not one for a universal rule. Those who desire to do so must be at liberty to continue as they began; those who wish for a different scheme must be free to agitate for it. To say that some great principle of professional reward or dignity is at stake is obviously absurd, for the voluntary practice has been in operation since the opening days of the war, and this without any serious challenge or discontent.

But now comes the point of disturbance. Gradually it has leaked out, in spite of official evasions and dexterities excessive even for the War Office, that the military authorities have the potential right to make certain payments to the medical officers of the V.A.D. hospitals. Not only so, but such payments have actually been made to certain doctors, who, somehow or other, discovered the secret. One of the conditions was that personal application should be made, and there was something more than a hint that the transaction must be a strictly private one, the recipient being bound in honour not to betray his good luck to his less well-informed colleagues. Naturally the discovery of this paltry and ridiculous proceeding has raised a storm of protest, and if now the authorities have to support widespread claims they will only have themselves to thank for the experience. Instead of a frank statement of the position and a proclaimed readiness to meet the position of men whose financial position prevented a continuance of contributory service, the officials concerned cultivate a kind of clever meanness, which, in the end, brings to them derision, if not contempt. It is hardly surprising that in such circumstances some members of the profession are inclined to hit back, and are tempted to say that if they are to be treated to secret huckstering they will, when the discovery is made, demand the full measure of their rights. Thus for a petty and tortuous economy there is incurred a risk of forfeiting the good will and confidence of a profession whose most cherished tradition is generosity of service. It is hardly possible to speak of such blundering with excessive severity.

But we repeat that these secretive and contemptible proceedings of the official world do not touch the position of the doctors relative to the V.A.D. hospitals at which they are serving. Each one of the practitioners concerned entered into an individual arrangement, and he is free to continue this arrangement or, on notice, to withdraw from it, as he deems necessary or advisable. What we feel quite sure of is that because some doctors now, perhaps quite justifiably, desire to be paid for their attendance, this will not be allowed to force the hands of others who propose to continue the original arrangement. The question is not one of principle but of individual convenience and circumstance, and everyone must be free to follow his own course. We quite agree that retrospective payments cannot be demanded. Men have stood in the public eye as voluntary workers and they cannot now claim the benefits due to paid officials. On the other hand, it is absurd for members of the public to declaim against men who after two to three years of skilled service voluntarily given feel themselves obliged to say that without some measure of remuneration they cannot afford any longer to sacrifice their personal interests. Some very exaggerated sums have been mentioned in the press, but the maximum sum paid to a civilian medical officer by the War Office is £1 per day.

